# Evolution and diversification of the nuclear envelope

**DOI:** 10.1080/19491034.2021.1874135

**Published:** 2021-02-09

**Authors:** Norma E. Padilla-Mejia, Alexandr A. Makarov, Lael D. Barlow, Erin R. Butterfield, Mark C. Field

**Affiliations:** a Division of Biological Chemistry and Drug Discovery, School of Life Sciences, University of Dundee, Dundee, UK; b Institute of Parasitology, Biology Centre, Czech Academy of Sciences, České, Czech Republic

**Keywords:** Nuclear envelope, heterochromatin, lamina, proteome, evolution, eukaryogenesis

## Abstract

Eukaryotic cells arose ~1.5 billion years ago, with the endomembrane system a central feature, facilitating evolution of intracellular compartments. Endomembranes include the nuclear envelope (NE) dividing the cytoplasm and nucleoplasm. The NE possesses universal features: a double lipid bilayer membrane, nuclear pore complexes (NPCs), and continuity with the endoplasmic reticulum, indicating common evolutionary origin. However, levels of specialization between lineages remains unclear, despite distinct mechanisms underpinning various nuclear activities. Several distinct modes of molecular evolution facilitate organellar diversification and  to understand which apply to the NE, we exploited proteomic datasets of purified nuclear envelopes from model systems for comparative analysis. We find enrichment of core nuclear functions amongst the widely conserved proteins to be less numerous than lineage-specific cohorts, but enriched in core nuclear functions. This, together with consideration of additional evidence, suggests that, despite a common origin, the NE has evolved as a highly diverse organelle with significant lineage-specific functionality.

## Introduction

Eukaryotes are estimated to have arisen over one and a half billion years ago – an event considered to be one of the major evolutionary transitions [1–3]. A consensus model is emerging [2], with the first eukaryotes likely evolving from within the Thaumarchaeota, Aigarchaeota, Crenarchaeota and Korarchaeota (TACK) superphylum [4]. Advances in environmental sampling and sequencing have superseded earlier rRNA-based models, which proposed three domains of eukaryotic life [5], to firmly place eukaryotes as emerging from within the Archaea and, hence, supporting a two domain paradigm [2]. At some point following the divergence from the archaeal lineage, the protoeukaryote began to acquire features typical of eukaryotic cells, including a tubulin-based flagellum, an endomembrane system, a cytoskeleton and the mitochondrion. Remarkably, this period of evolution produced a complex Last Eukaryotic Common Ancestor (LECA) that possessed a large diversity of compartments exceeding the complement of many extant unicellular organisms.

The fossil record is poor for this early period of evolution and preservation of cellular structure is of insufficient quality to draw unequivocal conclusions concerning the internal morphologies of early eukaryotic cells [6]. Consequently, molecular reconstruction of the evolutionary history of protein families is the major strategy employed to identify and, where possible, to order events during eukaryogenesis. The mitochondrion and the chloroplast are derived from endosymbiotic events and hence represent a distinctive evolutionary path [2,7]. The remaining structures/organelles in eukaryotic cells, including the nucleus, are considered to be endogenously-derived, i.e. arising through evolution and expansion in the gene complement of the proto-eukaryote; hence, the proteins defining various organelles have clear vertical descent from an archaeal ancestor. Reconstruction of the evolution of endogenously-derived organelles thus relies on the identification and analysis of marker genes/proteins that define specific organelles and their sub-structures.

**Figure 1.**
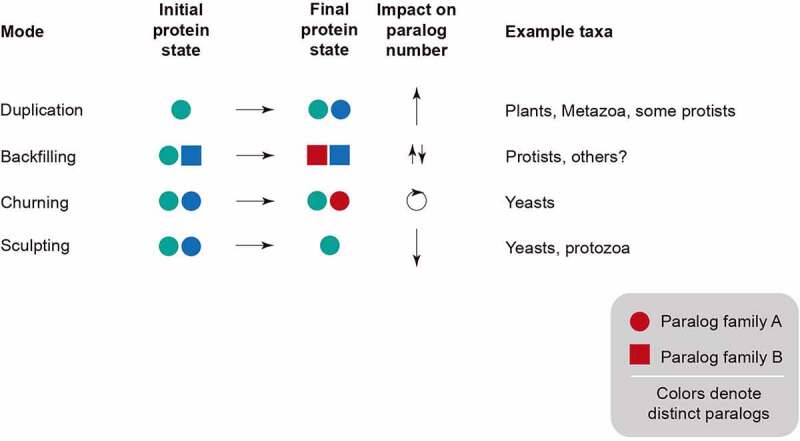
**Modes accompanying organelle evolution**. Multiple modes have been suggested for the evolution of paralogous genes involved in specifying organellar identity (see text). Gene duplication increases the number of paralogs, while sculpting decreases through gene loss. Churning can operate without an obvious or significant change to paralog number but represents the birth and death of paralogous, functionally equivalent genes while backfilling is the result of both gene loss and compensatory expansion of an unrelated gene family to suppress the impact of the initial loss

Several distinct modes underpinning the evolution of compartments and organellar diversity have been proposed. These are based largely on analysis of the membrane trafficking system, which features large paralogous protein families with organelle-specific members such as GTPase and SNARE families, kinesins and others [8–10]. Some of these modes can be considered to be ‘expansive’, in the sense that they lead to an increase in the number of compartments. This is exemplified by Rab GTPases that specify and mediate cargo exchange between endocytic and exocytic compartments [8]. Alternatively, there are many examples of reductive processes, where lineages lose complexity; again, the Rab family is a good example – in many unicellular organisms, for example in *Saccharomyces cerevisiae*, a great many Rab genes have been lost. We termed this process ‘sculpting’, as the new organism is revealed only through the removal of genes. These two examples are comparatively simple to understand in evolutionary terms as they rely on changes to the numbers of paralogs within a gene family [11]; but additional mechanisms are also at play and are not always obvious in terms of simple gene counts. These include ‘churning’ whereby paralogs are created and deleted so the overall number remains similar [12]; and finally ‘backfilling’ whereby loss of functionality through gene loss is compensated through the expansion of a different gene family [13] (Figure 1). In principle, the relevance of such modes of evolution extends to all endomembranes, including their sub-domains and functions, at all stages of eukaryotic evolution.

**Figure 2.**
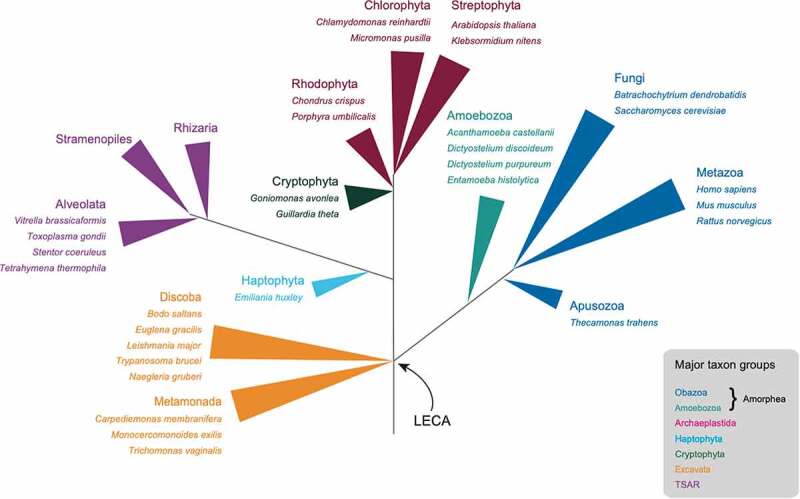
**Eukaryotic lineages sampled and their taxonomic relationship**. A schematic phylogenetic tree depicting the relationships between the major eukaryotic taxa. large groupings are color-coded as indicated in the legend at lower right and selected species included in the study are shown. LECA indicates the position of the last eukaryotic common ancestor. the tree structure is loosely based on the recent topology described in Burki et al., 2020

The nucleus is an endomembrane compartment with deep evolutionary connections to other endomembranes. In particular, nuclear pore complex (NPC) proteins and vesicle coat proteins mediating endocytosis, post-Golgi transport, ER-exit and flagellar assembly all have a common origin (reviewed in [14]). Despite significant variation in composition, the secondary structure of many NPC and coat proteins is well conserved [14], compelling evidence that the endomembrane system, and hence the great majority of the internal structure of the eukaryotic cell was well developed prior to the radiation of eukaryotes from the LECA (Figure 2). However, the details of several relevant events leading up to the LECA remain unresolved, including the relative sequential order of the origin of the nucleus and of the mitochondria [15,16]. For example, we have argued that the NPC, in its current configuration, likely emerged late in eukaryogenesis, although this does not preclude an earlier origin for the nuclear envelope in some form [17].

The nucleus facilitates separation of transcription and mRNA processing from translation and in modern eukaryotes a considerable machinery is devoted toward securing accurate mRNA processing prior to export from the nucleus [18]. Much of the apparatus is conserved, indicating ancient origins and a central requirement to minimize accumulation of aberrant proteins. Separation of translation and transcription enabled evolution of mRNA splicing, allowing greater functional diversity to be extracted from essentially the same coding sequences and providing additional mechanisms for controlling gene expression. Further complexity of gene regulation then arises from the development of sophisticated genome packaging that allows formation of eu- and heterochromatin and, further, to chromosomal territories and complex spatial genome organization. Some nuclear functions are potentially very ancient and are likely repurposed components from preexisting systems, for example, the ESCRT system involved in NE repair likely originated as a cytokinesis system in Archaea [19,20].

Despite these examples, variations in mechanisms of nuclear processes between organisms are well known. For example, nuclear envelope morphology during mitosis is usually either distinctly open or closed but can be semi-closed in some species such as *Schizosaccharomyces japonicus* [21]. These variations necessitate distinct mechanisms for segregation of nuclear components during division and reassembly. Open mitosis likely arose on multiple occasions, while closed mitosis is probably the ancestral as evidenced by the broader distribution of this mode [22]. Further, the mechanisms by which chromosomes are segregated are highly variable. While mitosis nearly exclusively requires a microtubule-based spindle, the location of the spindle anchor varies considerably, being at turns within the nucleus, embedded in the NE or in the cytoplasm [23]. Nuclear positioning and mechanotransduction, which in metazoa is mediated by the LINC complex, is also likely divergent; in vascular plants, there is a range of additional systems mediating cytoskeletal connections, while some organisms lack the LINC complex altogether [24,25]. Moreover, the nuclear lamina, a fibrous network residing on the nucleoplasmic surface of the NE, is represented by several apparently diselected for investigation. Givenstinct systems; NMCP/CRWN proteins of plants, lamins in many lineages including metazoa and NUP-1/2 of the kinetoplastida [25–29]. So far, mammalian lamin orthologs have not been detected in yeasts, either experimentally [30] or *in silico* [25]. However, in spite of lacking canonical lamins, yeast NPC components, nucleoporins and NE-associated proteins provide a platform for anchoring chromatin to the NE and for events related to gene expression regulation, transcription, mitosis and NE stability [31–33]. Lamins are likely the ancestral lamina proteins while the more restricted plant and kinetoplastid systems appear to be lineage specific and likely later emerging [25]. Finally, the kinetochore in kinetoplastids is composed of a distinct set of proteins differentiating these organisms from all other eukaryotes [34,35].

To extend our understanding of the dynamic process of nuclear evolution beyond the NPC and lamina to the whole of the NE, we examined a set of NE and NE-associated proteins, that we collectively term ‘NEA’, from metazoan and kinetoplastid datasets together with published work (Figure 2). We analyzed conservation of NEA proteins across eukaryotes, finding only a small contingent to be broadly conserved with the majority of the cohort being remarkably divergent; frequently, identifiable orthologs can only be found in closely related organisms.

## Methods

*Genome databases*. Eukaryotic species were selected for based on factors including importance as experimental organisms, phylogenetic diversity and sequence/assembly quality. This included recently published sequence data from the protozoan alga *Euglena gracilis* [36] and the freshwater sponge *Ephydatia muelleri* [37]. Details regarding all genomic and transcriptomic data sources are provided in Table S1. Although some datasets are from genome sequencing studies and some from transcriptomic studies, in each case only predicted peptide sequences were analyzed herein. In the case of *Carpediemonas membranifera* [38] and *Hemimastix kukwesjijk* [39], nucleotide transcript sequences were translated to predicted peptide sequences using GeneMarkS-T [40].

*Selection of genes for analysis*. Inclusive sets of NE and NE-associated (collectively NEA) proteins were selected for investigation. Given issues concerning with purity of subcellular fractions, we sought to minimize likely contaminants within NE proteome databases and no selection based on function was made. First Seventy-five relatively high-confidence rat liver NEA protein-encoding genes were selected from a much larger cohort of potential NEA proteins identified previously [41,42]. These were not limited to nuclear envelope transmembrane (NET) proteins and both inner and outer nuclear envelope proteins were included; importantly many have been identified at the NE of mammalian cells in subsequent studies and we excluded proteins subsequently demonstrated to have no NE-association [43–45]. Second, eighty-nine *Trypanosoma brucei* NE protein-coding genes were selected based on previous studies [46,47]. In this case, proteins containing at least one predicted transmembrane domain were included. TMHMM version 2.0 c transmembrane domain predictions from TriTrypDB were used for this purpose (accessed April 2020) [48,49]. We chose this selection criterion to maximize the likelihood that the included proteins were indeed associated stably with the NE and not a peripheral interactor.

*Comparative genomics*. Sequence analyses were performed on the University of Dundee High Performance Computing Cluster. Predicted peptide sequences of selected genes encoding nuclear envelope proteins were used as queries for searching across eukaryotes using the Basic Local Alignment Search Tool for Proteins (BLASTp, version 2.9.0+) [50] with an E-value <0.1. The top three hits per query per organism were extracted and further filtered by an alignment coverage of greater than 10% of the query sequence. Redundant, 100% identical sequences were not included in the hit count. Reverse BLASTp of hit sequences against the original query organism was performed and orthology predicted if the original query was within the top three hits, had an alignment coverage of greater than 10% and an E-value <0.1.

*Alignment and phylogenetic analysis*: For each query, sequences meeting the search criteria described above were aligned using MUSCLE [51] version 3.8.1551 with default settings. Alignments were trimmed using alncut version 1.06 [52] with gaps allowed in 25% of sequences per residue. Trimmed alignments were analyzed using FastTree version 2.1.10 [53] using default settings. In general, branch supports ≥0.95 were considered significant support for bipartitions.

*Validation and binning*: All data were manually inspected and met the following additional criteria beyond being included as a BLAST hit: (i) the length of the predicted protein is consistent with the query protein, (ii) the alignment demonstrates extensive regions of homology, and (iii) based on phylogeny, the putative ortholog is included in a monophyletic clade with the query and other orthologs (when paralogs were present). In many cases, the inclusive search criteria retrieved paralogs, which were excluded at this phylogenetic analysis step. Trees were viewed on Apple computers (macOS 10.15.7 operating system) using FigTree version 1.4.4 (http://tree.bio.ed.ac.uk/software/figtree) or the Environment for Tree Exploration (ETE3) version 3.1.2 [54], and alignments were viewed using AliView version 1.26 (http://ormbunkar.se/aliview) [55] or JalView version 2.11.0 [56]. In cases of ambiguity, bespoke BLAST searches at NCBI were used to further validate a conclusion of found/not found. Sequence IDs for all identified orthologs are provided in Table S2.

Search results were binned according to inferred timing of evolutionary origin: Either originating in the LECA (Group A), in an amorphean ancestor of Metazoa (Group B), or much later within either Metazoa or Euglenozoa (Group C). Results in Group A included both those with orthologs nearly universally conserved among eukaryotes and those with orthologs not identified in several major taxonomic groups. Data from comparative genomics searches were converted to the Coulson plot format using CPG version 1.6.1 (https://github.com/mfield34/CPG [57]) and finalized using Illustrator (https://www.adobe.com).

*Gene ontology*: Gene ontology annotation for Biological Processes for mammals was obtained from Uniprot (https://www.uniprot.org) [58] and/or Quickgo-EBI (https://www.ebi.ac.uk/QuickGO/) [59] using accession IDs. Gene Ontology annotations were categorized and frequency was represented in bar charts using Microsoft Excel and finalized using Adobe Illustrator. Gene ontology annotation for trypanosome proteins were retrieved from TryTripDB release 48 beta ([49], https://tritrypdb.org/tritrypdb/app).

## Results and discussion

*Documenting nuclear envelope evolution and understanding eukaryogenesis*. In the earliest stages of eukaryotic evolution, the nucleus may have served as a simple membranous structure enclosing the chromatin. The double lipid bilayer and composition of the NE are direct consequences of the NE being an extension of the endoplasmic reticulum (ER). With several nuclear and NE proteomes published [41,60–62], data suggest retention of functional analogs, *albeit* with divergent sequence and structure, but most information is restricted to animals and fungi. Further, protein domain shuffling [63,64] is also apparent among the NEA proteins, for example, the lamin B receptor is conserved but only due to the presence of a C_14_-sterol reductase domain, indicating repurposing but not a pan-eukaryotic presence for an LBR [25]. Hence, more systematic analysis is warranted.

We explored NEA protein conservation by conducting searches for orthologs of 164 putatively NEA genes, using reciprocal-best-hit searching with BLAST. This cohort included 75 genes originally identified in mammals [41–45] and 89 proteins identified in *Trypanosoma brucei* [47]. The data returned from this procedure (supplementary data archive) were manually validated. All calls are conservative, with only orthologs detected with confidence deemed ‘found’. Our search included members of the Amorphea (comprising organisms belonging to Opisthokonta, Amoebozoa and Apusomonada supergroups), Archaeplastida (including a diversity of plants and members of Chlorophyta and Rhodophyta), Cryptista (represented by members of Cryptophyta), Excavata (flagellated organisms of biological and/or medical importance such as *T. brucei* and *Leishmania major*), CZAR (which encompasses several major groups of algae, protozoa and seaweeds), Haptista (comprising haptophyte algae, represented in this work by the marine microalgae *Emiliania huxleyi*) and Hemimastigophora (an early-branching lineage of free living protozoa with two rows of flagella, represented here by *Hemimastix kukwesjijk*) [39,65]. All genomes were selected for quality in terms of predicted proteome coverage completeness (see Methods for data sources and Figure 2 for taxonomic context).

**Figure 3.**
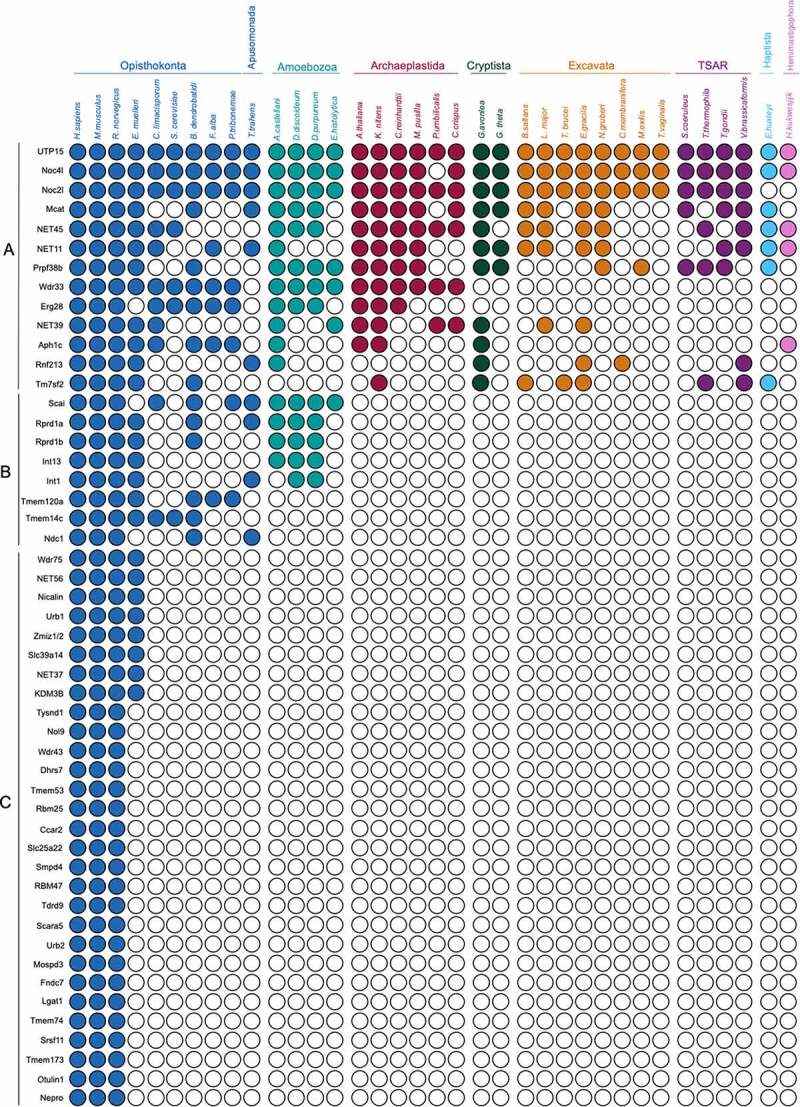
**Distribution of nuclear envelope proteins from metazoa across eukaryotes**. Coulson plot demonstrating presence or absence of NE proteins across the eukaryotes. Filled circles indicate proteins identified, open circles indicate proteins for which orthologs were not found. Rows are predicted proteins and columns are organisms. Supergroups are colourised using the same system as in Figure 2. Three groups are recognized: Group A; highly conserved across Eukaryotic supergroups, Group B; originated in an amorphean ancestor of Metazoa

**Figure 4.**
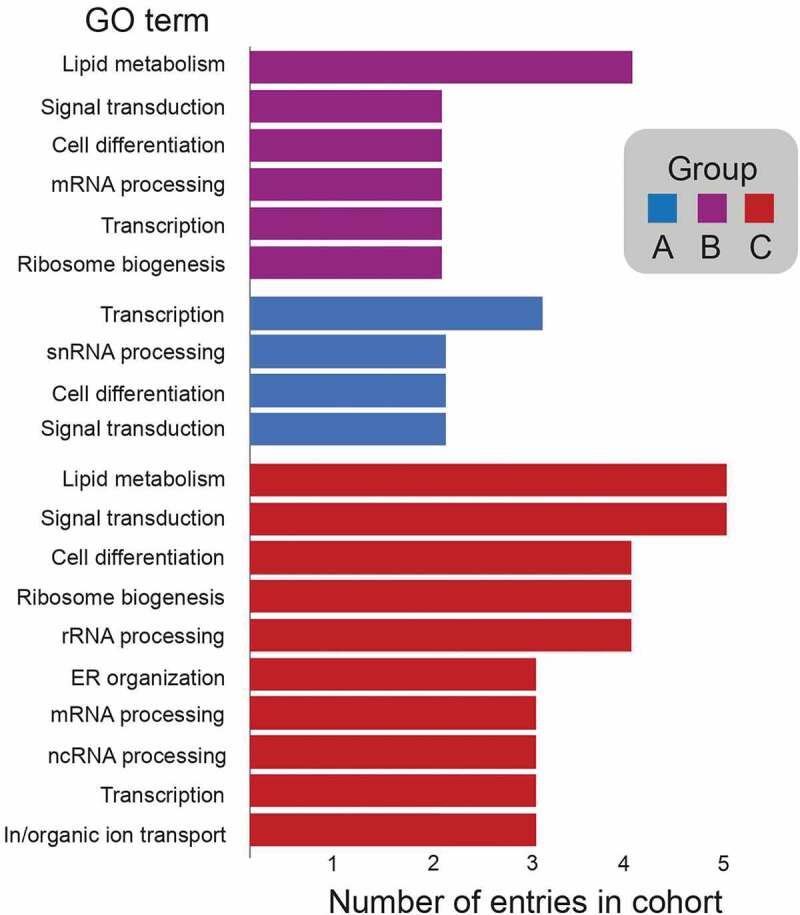
Frequency of gene ontology annotation for nuclear envelope proteins from the metazoan cohort. bar chart showing frequency of GO annotation of Biological process of highly conserved proteins across supergroups (Group A), proteins present across Amorphea (Group B) and proteins restricted to Metazoa (Group C) from .Figure 3

*Lineage-specific proteins dominate metazoan NEA protein cohorts*. Firstly, a cohort of 50 NEA proteins (Figure 3) identified initially in rodents [41] and subsequently in several human cell lines [42–45] were used as queries. We distinguished three groups (see Methods). Group A (13 proteins) are highly conserved across eukaryotes indicating an origin predating the LECA, and thus likely to support universally required functions. Gene ontogeny (GO) analyses for Biological Process revealed that these proteins are mostly involved in lipid metabolism (Figure 4), consistent with this interpretation. Group B (eight proteins) includes proteins present only in amorphean groups, i.e., Metazoa (including mammals), Fungi, Apusomonada, and Amoebozoa. GO identified these proteins as mainly related to transcription. Many of Group B may have been lost due to saprophytic or parasitic lifestyles in which the host is able to provide these requirements, as indicated on the specific losses within the Amorphea that collectively exemplify sculpting. Group C (29 proteins) comprises proteins restricted to Metazoa, suggesting an origin in a recent ancestor. NE and membrane (NE and/or ER) associated proteins in this group partake in a variety of general functions and we observe an enrichment in proteins involved in the lipid metabolism, RNA processing (including ribosomal, messenger RNAs and non-coding RNAs such as t-, sn- and piwi-RNAs) and ion transport. Additionally, several proteins stand out as being involved in complex processes characteristic to metazoans: in innate immune response, such as Tmem173/NET23/STING [66,67], and tissue differentiation and development, such as NET37 in skeletal muscle development [68], Mospd3 in heart development [69], Nepro in neurogenesis [70]; and multiple other proteins with tissue specific functions at the nuclear envelope that appear critical for healthy tissue maintenance (Wolframin, Tmem201, Emerin and Nesprin/SYNE proteins, see below). This participation of the NEA proteins in cell/tissue differentiation is commonly accompanied by functions in signal transductions, highlighting the importance of this set of proteins in triggering signaling pathways such as MTOR, Wnt, MDA-5, Notch, kinase cascades, etc. Moreover, transcriptional regulation is a common function in the cohort, influencing regulation of RNA polymerases (I and II) such as Noc2l which acts as a transcription corepressor [71], Wdr43 which is needed for activation of promoters and favors transcription elongation [72], Int1 [73], Zmiz1/2 [74] and Rprd1b/1a which are transcriptional regulators of RNA polymerase-II transcription [75,76].

Importantly, targeting to the NE of many of these proteins has been verified experimentally as, in spite of having at least one TMD, some of these proteins do not localize to the NE but rather to the nucleoplasm or nucleolus [77]. Interestingly, some NEA proteins can be detected by microscopy at the nuclear periphery in some cell types/tissues but not in others, which has led to the concept that different tissues contain distinct pools of protein complexes that alter the localization of NEA proteins [77], making prediction of NE localization even more challenging. Although most of the proteins in our cohort reside in the NE, some of them have shown nucleoplasm localization, such as UTP15, Noc4l, Noc2l, Wdr33, Wdr75, Tmem74 [43,77]. Interestingly, some NEA proteins have been localized in different regions of the nucleus, e.g. lamin A, which typically localizes to the nuclear rim but in recent years a highly mobile nucleoplasmic pool of lamin A has been detected [78–80], although the mechanisms regulating this state of lamina-independent lamins remain obscure. Further examples are Tmem214 and Tmem70: Tmem214 is simultaneously identified as a protein [81], an ER transmembrane protein [82] and further confirmed to localize to the inner nuclear envelope [83]; Tmem70 has multiple isoforms and is traditionally considered an inner mitochondrial membrane protein partaking in the assembly of the ATP synthase [84,85], but has also been identified as a NET [83].

Thus, association of proteins with the NE seems to be complex to predict merely by bioinformatic tools, as their localization is influenced not only by structure but also by interacting partners and differential expression in different cell types (see below) and the orthologs of mammalian NEA proteins detected here will require experimental validation of NE association in other organisms.

**Figure 5.**
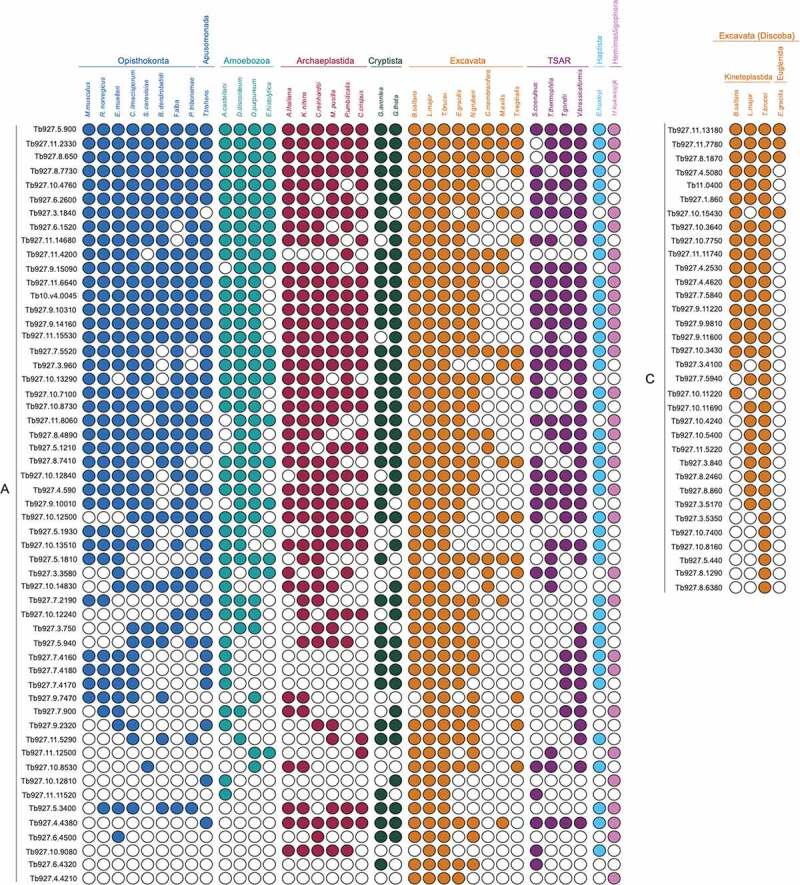
**Distribution of trypanosomatid nuclear envelope proteins across eukaryotes**. Coulson plot demonstrating presence or absence of trypanosome NE orthologs across the eukaryotes. filled circles indicate proteins identified, open circles indicate proteins for which orthologs were not found. rows are predicted proteins and columns are organisms. supergroups are colourised using the same system as Figure 2. Two groups are recognized: Group A; scattered distribution across taxa and Group C; restricted to kinetoplastids. TriTrypDB [49] accession IDs are shown on the left

*Lineage-specific NEA proteins are widespread*. Metazoan sequences cannot be used to identify non-orthologous lineage-specific genes in other taxa, and also have inherent bias toward metazoa-specific genes [86]. Hence, secondly, we searched using queries from the protozoan *T. brucei* [47] (Figure 5). Overall, trypanosome Group A (55 proteins) appears to have core biological functions, with GO annotation (Biological Process) indicating involvement in inorganic and organic ion transport, protein glycosylation and lipid metabolic process (Table S3). Orthologs of some trypanosome Group A proteins were readily identified in all or nearly all other eukaryotes. For example, Tb927.5.900 is an oligosaccharyltransferase subunit, with orthologs retrieved in all genomes queried in our analysis. In other eukaryotes, these are essential for transfer of N-glycans [87] and localize at least in part to the NE [88]. Some Group A proteins, however, were less conserved, for example, the transmembrane protein Tb927.10.12810 which contains a choline phosphate cytidylyltransferase domain. In most eukaryotes, choline phosphate cytidylyltransferases localize to the NE and regulate phosphatidylcholine levels [89]. However, outwith kinetoplastids, orthologs of Tb927.10.12810 were only identified in organisms representing the unicellular taxa Apusomonada, Amoebozoa, Cryptophyta, and Hemimastigophora (Figure 5).

Group C (34 proteins) includes several proteins associated with lipid biosynthetic processes (e.g., sphingolipid, fatty acid biosynthesis) and proteins targeting to the ER; however, majority of proteins in this group are annotated as hypothetical or putative gene products. Several proteins are trypanosome-specific, represented by *T. brucei* in our analysis, including a putative *trans*-sialidase (Tb927.5.440), a SUMO-interacting motif containing protein (Tb927.8.1290) and three hypothetical proteins (Tb927.3.5350, Tb927.8.6380 and Tb927.10.8160 with experimental evidence of localization to the NE and ER [49,62,90]. Overall, the frequency of Euglenozoa-specific proteins is comparable to the results of searches using metazoan NEA protein queries.

**Figure 6.**
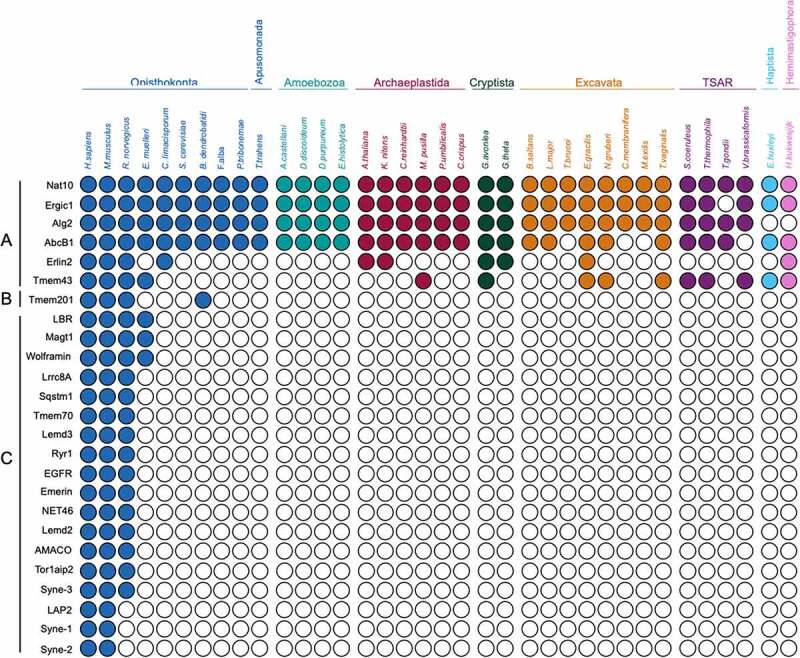
**Distribution of disease-associated nuclear envelope proteins across eukaryotes**. Coulson plot demonstrating presence or absence of NETs directly linked to human disease. Supergroups are colourised using the same system as Figure 2, Layout as in Figure 3. Three groups are recognized: Group A; highly conserved across Eukaryotic supergroups, Group B; originated in an amorphean ancestor of Metazoa, Group C; restricted to Metazoa. See text for explanation as to why LBR is excluded from Group A

Moreover, with a single exception, metazoan queries (Figure 3) did not retrieve any proteins from our trypanosoma query cohort (Figure 6) but instead others that may be important as NEA proteins, namely a beta propeller protein Tb927.8.1980, a Noc2p family protein Tb927.10.12430, CBF/Mak21 family protein Tb11.v5.0274/Tb927.10.6320/Tb927.11.2120/Tb927.4.3670. The exception to this lineage-specificity is Tm7sf2 (E9Q4M8), which appears to be an ortholog of *T. brucei* Tb927.11.15530 (Figures 3 and 6). *Frequency of lineage-specific proteins in fungi and plants*. To expand our coverage, we also examined published analysis of NE proteomes in both yeast and plants. An earlier systematic study focused on forty-five budding yeast proteins selected for NE localization from a high throughput dataset [91]. In this case ~50% of the cohort were suggested as pan-eukaryotic with a further ~20% broadly distributed. The conserved proteins were in the main involved in core nuclear functions, including chromatin organization, with only 20% predicted as unique to Saccharomycetes. A considerable number of homologs could only be identified through a shared domain, indicating possible domain swapping as noted above for the LBR. This pattern may in part be a reflection of the extensive gene losses in *S. cerevisiae*, such that lineage-specific proteins are depleted. A high incidence of domain sharing amongst NEA proteins from different lineages, but within otherwise highly divergent proteins, is also supported by evidence from the vascular plant *Arabidopsis thaliana*. The *A. thaliana* LINC complex for example is canonical, containing both SUN and KASH-domain proteins. However, the nesprins (see below) of metazoan cells, which also contain KASH domains and associate with the core LINC complex, are replaced by analogs that only retain a KASH domain but no other obvious homology [24,92]. Given that the KASH domain is quite short (less than 60 amino acids and in plants frequently even shorter) how this system has evolved is intriguing. SINE proteins are present across a large number of vascular plants and are components of the analogous LINC complex and likely support a link between the plant-specific lamina NMCP/CRWN proteins and the cytoskeleton [93]. There is considerable promiscuity here as all SINE proteins interact with SUN1 and SUN2; this may be related to the manner in which this family has expanded, with clear ancestral as well as lineage-restricted members that presumably evolved later. This may be, at least in part, due to tissue-specific expression [93].

*NEA disease genes are dominated by lineage-specific members*. The relevance of NEA proteins to a range of diseases is well documented [94–96]. To uncover whether disease-associated NEA proteins are involved in metazoan-specific or general eukaryotic functions, we examined the distributions of 25 NEA proteins previously linked to pathology (Figure 6) and encompass a broad range of conditions including myopathies, γ-globulinemia, inflammatory diseases, muscular/skeletal abnormalities, cardiomyopathies, neurological/mental conditions, glycosylation disorders and others ([42] and references in Table 1). The ability to identify an ortholog in another organism could, for example, be exploited in determining function. Nat10, Ergic1, Alg2 and Abcb1 are present broadly in all supergroups explored (Figure 6, Group A). These proteins relate to near universal functions such as maturation of SSU-rRNA, vesicle mediated transport and ATP transport, respectively. However, although related to disease, mutations in these genes do not necessarily result in detrimental phenotypes in other eukaryotes. This is reflection of context dependence, and we have noted this previously regarding some essential genes in metazoa which are absent from multiple unicellular taxa [86]. We found Alg2, Erlin2 (ER lipid raft-associated 2) and Tmem43 retained in several taxa. Mutations in Tmem43, a four transmembrane domain protein known as LUMA in *Homo sapiens*, are linked to cardiac conduction defects [97] and Emery-Dreifuss muscular dystrophy [98,99]. Moreover, Tmem43/LUMA upregulation is related to poorly fertile spermatozoa [100] and Tmem43/LUMA  has close interactions with lamins and emerin and is involved in structural organization of the NE [99,101]. Erlin2 is a transmembrane domain protein involved in lipid homeostasis [102] and leads to motor and cognition disabilities when mutated [103]. Moreover, abnormal Erlin2 levels are found in human breast cancer [104]. Alg2, a mannosyltransferase, is involved in N-glycosylation and associated with glycosylation disorders (Table 1 and references therein). One protein seems to have originated earlier in Amorphea, Tmem201 (Figure 6, Group B), which contributes to the architecture of the NE and interacts with the LINC complex through UN1 and lamin A [105]. Mutations in this protein have been recently implicated to cause one of the variants of Emery-Dreyfuss muscular dystrophy [106].Interestingly, the eighteen remaining disease-related proteins are apparently metazoa-specific, (Figure 6, Group C, Table 1). Lamin B receptor (LBR), one of the better characterized NEA proteins residing in the inner nuclear membrane, is an important interactor of lamin B [107]. With lamina-constituting proteins being divergent in different systems [25], lamin B receptor may also constitute a case of divergence across supergroups despite its C_14_-sterol reductase domain having a pan-eukaryotic distribution and influencing metazoan cell viability and embryogenesis in plants [108–110]. Although several proteins are associated with specific diseases, such as Nesprin 1 and 2, emerin and lamin A-associated polypeptide 2 (LAP2) (Emery-Dreifuss dystrophy and cardiomyopathy) [98,111]; others have broad pathology, such as Sqstm1 (Sequestosome 1), associated with bone, muscle and heart defects, neurodegenerative conditions and leukemia, or EGFR (Epidermal growth factor receptor) related to cancer and bowel disease (Table 1). Nesprins (Syne-1, 2 and 3) localize at the NE and are scaffolding proteins that partake in the LINC complex [112,113]. Besides nuclear structure, emerin also has roles in transcription, signaling and chromatin regulation through interactions with partner proteins [114]. It is interesting that the distributions of emerin and Nesprin-1/2/3 are substantially narrower than that of the LINC complex – defined as SUN/KASH-domain protein complex crossing the nuclear lumen. As remarked above, this is likely due to replacement with functional analogs. *Tissue-specificity of nuclear envelope proteins*. Hundreds of mutations are known in genes encoding NEA proteins, including in lamins, nuclear pore components and NETs (Table 1). Although most of these proteins are ubiquitously expressed, the mutations are associated with a wide number of diverse and apparently unrelated tissue-specific diseases. Further, in many cases a single protein can harbor several mutations each causing a different disease, e.g., Lbr where mutations can cause bone disease or blood disorders (Table 1 and references therein). One prevalent hypothesis for how this occurs is that specific pathology develops from disruption of a tissue-specific function, for example, interaction with a tissue-specific partner. In Table 2, we summarize recent findings on the differential expression of proteins present in the cohorts in Figures 3 and 6.NE proteins can influence chromatin topology and gene expression, for example, Tmem201 (NET5), Tmem120A (NET29), NET45, NET39 and Tm7SF2 (NET47), which promote peripheral positioning of chromosomes [115–117]. Different NETs demonstrate differential impact on chromosomal positioning [116] and some, e.g. Sting1/NET23, alter chromatin through generating changes in the chromatin compaction [67]. Moreover, NEA proteins also influence transcription, e.g. Tm7SF2 during hepatic differentiation, or act as repressors as in the case for NET39, Tmem38A and WFS1 in muscle cells [115]. These activities of gene repositioning are critical for differentiation, e.g. Tmem120A in adipocytes, Tm7SF2 in hepatic cells and NET39, Tmem38A and WFS1 in myogenesis [67,115,117]. Tmem201 (Figure 6, group B), Tmem120A (Figure 3, group B) were found restricted to Opisthokonta, and Tm7SF2 (Figure 3, group C) was restricted to Metazoa, while NET45 and NET39 were identified broadly (Figure 3, group A). Whether NET45 and NET39 or other wider-distributed proteins can influence chromosome topology or induce gene regulation signaling pathways in single-cell organisms is yet to be explored.

**Table 1.** Diseases associated with nuclear envelope-associated genes restricted to Metazoa

Gene	Protein	Associated Disease	Reference
*Ergic1*	Endoplasmic reticulum-Golgi intermediate compartment protein 1	Arthrogryposis multiplex congenita (AMC, neurophatic type), gastric cancer	[119,120]
*Nat10*	N-acetyltransferase 10	Hepatocellular carcinoma HGPS (Hutchinson-Gilford progeria syndrome), Muscular dystrophy	[121–124]
*Alg2*	α-1,3/1,6-mannosyltransferase	Congenital disorder of glycosylation (type Ii), Congenital myasthenic syndrome	[125–127]
*AbcB1*	ATP-binding cassette subfamily B, member 1	Inflammatory bowel disease 13, Encephalopathy	[128,129]
*Tmem43*	Transmembrane protein 43	Ventricular displasia 5 (ARVC5), Emery-Dreifuss muscular dystrophy 7	[99, 130–132]
*Erlin2*	ER lipid raft associated 2	Spastic paraplegia 18, Motor system conditions	[103,133]
*Lbr*	Lamin B receptor	Greenberg skeletal displasia, Pelger-Huet anomaly, Reynolds síndrome	[134,135]
*Magt1*	Magnesium transporter 1	Immunodeficiency with magnesium defect, Epstein-Barr virus infection and neoplasia (XMEN), Mental retardation (X-linked), Congenital disorder of glycosylation 1CC (CDG1CC)	[136,137]
*Wfs1*	Wolframin	Wolfram syndrome (diabetes mellitus, optic atrophy, deafness)	[138,139]
*Tmem201*	Transmembrane protein 201	Emery-Dreifuss muscular dystrophy	[106]
*Lrrc8A*	Leucine rich repeat containing 8 family, member A	Agammaglobulinemia, Glaucoma	[140,141]
*Sqstm1*	Sequestosome 1	Paget disease (bone turnover), Dementia, Sclerosis, Neurodegeneration, Myopathy, Alzheimer’s disease, Lymphoblastic leukemia	[142–146]
*Tmem70*	Transmembrane protein 70	Mitochondrial disease (ATP synthase deficiency)	[147]
*Lemd3*	LEM domain containing 3	Buschke-Ollendorff syndrome (skin and bone disorders)	[148]
*Ryr1*	Ryanodine receptor 1	Malignant hyperthermia, Myopathy, Neuromuscular disorder	[149–152]
*EGFR*	Epidermal growth factor receptor	Lung cancer, Neonatal skin/bowel disease	[153,154]
*Emd*	Emerin	Emery-Dreifuss muscular dystrophy	[98,155]
*NET46*	Steroid transmembrane transporter	Connotruncal heart defects (CTDs) in newborns	[156]
*Lemd2*	LEM domain-containing protein 2	Juvenile-onset cataracts in Hutterties population	[157]
*AMACO*	A domain-containing protein similar to matrilin and collagen	Colon cancer	[158,159]
*Tor1aip2*	Torsin A-interacting protein 2	Dystonia	[160]
*LAP2*	Lamin A-associated polypeptide 2, Thymopoietin	Cardiomyopathy	[161]
*Syne-1*	Nesprin 1 (Nuclear envelope spectrine repeat protein 1)	Spinocerebellar ataxia, Emery-Dreifuss muscular dystrophy, Arthrogryposis	[98, 112, 162, 163]
*Syne-2*	Nesprin 2 (Nuclear envelope spectrine repeat protein 2)	Emery-Dreifuss muscular dystrophy	[98,112]
*Syne-3*	Nesprin 3 (Nuclear envelope spectrine repeat protein 3)	Emery-Dreifuss muscular dystrophy	[106]

**Table 2.** Differential expression in tissues of envelope-associated genes from Metazoan cohort

Proteins from Metazoan cohort (Figure 3)			
Group in Coulson plot	Protein	Expression in tissues^1^	Reference
A	NET45, Mcat, Wdr33, NET39, Slc25a22	Enriched in liver and blood.	[43]
	NET11	Enriched in muscle and blood (B-cells).	[43]
B	Scai	Widely expressed. Higher levels in brain, spleen and thymus.	[164]
	Rprd1b	Widely expressed. Higher levels in liver, colon, prostate, uterus. Lowest levels in heart and kidney. Not detected in rectum.	[165]
	Tmem120a	Widely expressed. Higher expression in liver, heart, kidneys, colon, nociceptors and adipose tissue.	[166,167]
C	NET56	Predominantly muscle specific, with lower expression in other tissues.	[168]
	NET37	Brain, liver, spleen, skeletal muscle, heart, lung and kidney. High protein levels in astrocytes and skeletal muscle.	[68]
	Nicalin	Enriched in liver and blood.	[43]
	KDM3B	Ubiquitous, highly expressed in placenta, skeletal muscle, kidney, heart and liver.	[169]
	Wdr43	Low levels in heart, liver, lung, spleen, thymus and hippocampus.	[170]
	Lpgat1	Highly expressed in liver and placenta. Expressed in peripheral blood, lung, kidney and brain. Lower levels in colon.	[171]
	Tmem173 (Sting1)	Highly expressed in mature B cells. Present in spleen, thymus. High protein levels in dendritic cells.	[172,43]
	Torp1aip2	Widely expressed. Higher levels in non-neural cells, hippocampus and spinal cord, liver and B cells. High levels of protein in liver.	[173,43]
	Tm7sf2, Scara5, Tmem74, Tmem53	Enriched in liver.	[43]
	Tmem173, Dhrs7	Enriched in blood.	[43]
	Mospd3	Enriched in liver and muscle.	[43]

Proteins from Diseases cohort (Figure 6)			
Group in Coulson plot	Protein	Expression in tissues^1^	Reference
A	Alg2	Enriched in liver and blood.	[43]
	ABCB1	Expressed in liver, kidney, small intestine, blood and brain.	[58, 43]
	TMEM43	Highest levels in placenta. Lower levels in heart, ovary, spleen, small intestine, thymus, prostate and testis.	[101]
B	Tmem201	Widely expressed.	[43]
C	LBR	Expressed in the bone marrow, liver, heart, adrenal gland, lung, placenta and uterus. Expressed in osteoclasts and osteoblast-like cells.	[174]
	MAGT1	Ubiquitous. Low levels in brain, lung and kidney.	[175]
	Wolframin	High levels in heart, followed by brain, placenta, lung and pancreas. Low levels in liver, kidney and skeletal muscle.	[58]
	LRRC8A	High levels in bone marrow. Also found in brain, kidney, ovary, lung, liver, heart, blood cells, T-cells and B-cells.	[140]
	SQSTM1, EGFR, Lemd3, Lemd2	Ubiquitous.	[176, 177, 45]
	RYR1	Found in skeletal muscle and brain.	[178]
	Tmem70	Enriched in blood and muscle.	[43]
	Emerin	Skeletal muscle, heart, colon, testis, ovary and pancreas.	[58]
	NET46	Highly expressed in kidney, enriched in liver.	[179, 43]
	Amaco	Detected in uterus, kidney, skin, lung, intestine.	[180]
	LAP2	Ubiquitous. Abundant in adult thymus and fetal liver.	[58]
	Syne3	Ubiquitous.	[44]
	Syne1	Ubiquitous. Highly expressed in skeletal and smooth muscles, heart, spleen, leukocytes, pancreas, cerebellum, stomach, kidney and placenta.	[181, 182]
	Syne2	Ubiquitous. Higher levels in kidney, adult and fetal liver, stomach, placenta. Low levels in skeletal muscle and brain. Isoform 5 highly expressed in the pancreas, skeletal muscle and heart.	[181]

^a^Refers to levels of mRNA otherwise stated.

## Conclusions

Reconstructions from comparative genomics have established an overall pattern of conservation of endomembrane-system genes among all major lineages of eukaryotes including plants, protists, amoebae, animals and fungi, implying inheritance from a complex common ancestor [14]. It also suggests that a widely conserved, complex set of NEA proteins may exist, corresponding to the conservation of the NE itself. While 300–400 putative NEA proteins have been described in human and yeast cells, few orthologs of these proteins have been described across eukaryotic diversity to date. While the NEA protein datasets upon which we based our analysis are of high quality, we must emphasize the need for further experimental and *in silico* work to define NEA protein cohorts with greater confidence. Inherent to any proteomics investigation are sources of error, including contaminants, mislocalized proteins and proteins evading detection by mass spectrometry. In the case of the NE, isolation of pure fractions for proteomic profiling is particularly challenging due to continuity of the NE with the ER, and due to fibrous connections between the NE and both the cytoskeleton and the nucleoplasm. Further complexities arise, as many NEA proteins have dual localizations at the NE and other organelles such as ER, cytoskeleton, mitochondria and peroxisomes, and some proteins are only identified in the NE while in the early stages of their biosynthesis [60,62]. For some proteins, NE functions may be carried out by a minor fraction of the total cellular pool, as some of them can be detected in different organelles or even multiple localizations within the same organelle. Moreover, although orthologs tend to retain function [118], many widely conserved NEA proteins are characterized in only one model organism, leaving their functions in distantly related eukaryotes uncertain. This, together with the apparent prevalence of lineage-specific NEA proteins revealed here warrants parallel experimentation efforts using multiple model organisms. Our present analysis is certainly not exhaustive and is intended to provide a broad overview of modes of NEA protein evolution. We are limited not only by uncertainty in experimental identification of NEA proteins but also sources of potential error inherent to sequence similarity search methods. For example, we are aware that highly divergent orthologs will not have been retrieved by our BLAST searches, but might be retrieved through further analysis with profile searching methods. Also, we have attempted to be conservative, preferring to exclude potential orthologs when there is reason for significant doubt. Thus, further analyses may reveal somewhat more (or less) extensive diversity in nuclear composition and hence, function. Certainly, examples of churning or backfilling would not be detected here. Nevertheless, considerable divergence in NEA protein cohorts between lineages is clear, with 50% or fewer proteins detected as broadly distributed, despite low stringency searching followed by manual validation. Ribosome biogenesis, RNA processing proteins and proteins with participation in transcription are highly conserved, unsurprisingly, followed by proteins involved in lipid biosynthesis [91]. Regardless, the cohort is dominated by lineage-specific proteins, many of which, like nesprins, possibly have analogs in other lineages. Hence, when considering the NE, variations in the NPC, lamina systems, cytoskeletal anchoring and kinetochores, the nucleus provides an example of considerable diversity despite hosting a plethora of core functions (Figure 7). Indeed, these findings suggest a rather surprising level of divergence associated with a structure that, in a very real sense, defines the eukaryotic cell.

## Supplementary Material

Supplemental Material
