# Supplementary material for: Evolution and diversification of the nuclear envelope
Source: Nucleus. 2021 Feb 9;12(1):21–41. doi: 10.1080/19491034.2021.1874135 (PMC7889174; doi:10.1080/19491034.2021.1874135)
Supplement: Supplemental Material [file KNCL_A_1874135_SM3076.zip › Supplementary information/Supplement material.docx]

**Supplementary material for:**

**Evolution and diversification of the nuclear envelope**

Norma E. Padilla-Mejia^1^, Alexandr Makarov^1^, Lael D. Barlow^1^, Erin R. Butterfield^1^ and Mark C. Field^1,2*^

^1^School of Life Sciences, University of Dundee, Dundee, DD1 5EH, UK and ^2^Institute of Parasitology, Biology Centre, Czech Academy of Sciences, 37005 České Budějovice, Czech Republic.

**Supplementary table legends**

**Table S1: Sources of genomic and transcriptomic data.** Source web addresses are provided for each set of sequences analyzed.

**Table S2: Sequence IDs for all identified orthologs.**

**Table S3: Gene ontology annotation for nuclear envelope proteins in *Trypanosoma brucei*.** Proteins are grouped as highly conserved proteins across supergroups (Group A), proteins present only in specific supergroups (Group B) and proteins restricted to kinetoplastida (Group C) from Figure 4.

**Supplementary data archive:** Alignments and phylogenetic analyses of identified homologs, and FASTA files with orthologous sequences identified for each query.
